# Flicker electroretinogram in preterm infants

**DOI:** 10.1038/s41433-024-03127-9

**Published:** 2024-05-23

**Authors:** Aylin F. Taner, James V. M. Hanson, Caroline Weber, Dirk Bassler, Daphne L. McCulloch, Christina Gerth-Kahlert

**Affiliations:** 1 Department of Ophthalmology, University Hospital Zurich and University of Zurich, Zurich, Switzerland; 2 Department of Neonatology, University Hospital Zurich and University of Zurich, Zurich, Switzerland; 3 School of Optometry and Vision Science, University of Waterloo, Waterloo, Canada

**Keywords:** Retinal diseases, Retina

## Abstract

**Background:**

Infants born prematurely are at risk of developing retinopathy of prematurity, which is associated with abnormalities in retinal function as measured using electroretinography. The aim of this study was to record non-invasive flicker electroretinograms (ERGs) in preterm infants and compare function of moderate and very or extremely preterm infants.

**Methods:**

In this non-randomized, cross-sectional study, 40 moderate preterm (gestational age (GA) 34 0/7 to 36 6/7 weeks, Group A) and 40 very or extremely preterm infants (GA ≤ 31 weeks, Group B) were recruited for flicker ERG recording through closed eyelids using the RETeval® device and skin electrodes. Group A was tested within the first week of life and Group B between 34th and 37th week postmenstrual age. Flicker stimuli were presented at 28.3 Hz with stimulus levels of 3, 6, 12, 30 and 50 cd•s/m^2^. Primary endpoints were peak time (ms) and amplitude (µV).

**Results:**

Flicker ERGs were recordable in most infants with the highest proportion of reproducible ERGs at 30 cd•s/m^2^. Amplitudes increased with stronger flicker stimulation, while peak times did not differ significantly between stimulus levels nor groups. Amplitudes were significantly greater in Group B at the strongest stimulus level (Mann-Whitney-U-Test=198.00, Z = 4.097, p = <0.001).

**Conclusions:**

Feasibility of collecting flicker ERG data in most preterm infants was confirmed. We found no evidence of reduced retinal responses to flicker stimuli associated with extreme prematurity. Higher amplitudes in very and extremely preterm infants could indicate acceleration of retinal development following birth, triggered by visual stimulation.

## Introduction

Between mid-gestation and the first postnatal months the retina matures rapidly, including growth of the retina with migration of inner retinal neurons towards the periphery, development and elongation of the photoreceptor inner and outer segments, and increasing rhodopsin content of rod cells. Thereafter, retinal development progresses more slowly until late childhood. At term birth, the retina of an infant shows significant immaturities [1–3]. Retinal vascularisation begins before the 15^th^ week of gestation at the central retina and extends toward the periphery until after term age [4]. In cases of premature birth, the complex interaction of neural and vascular tissue during retinal development takes place in a very different environment than in utero. This can lead to anomalous retinal development such as retinopathy of prematurity (ROP), which is characterised by abnormal retinal vasculature. Development of ROP is coincident with the rapid maturation of the retina before the preterm child reaches term age [5].

Electroretinography (ERG) is a widely used diagnostic tool to quantify retinal function by measuring electrical responses to light stimuli. ERG can measure retinal sensitivity and responsivity and hence document maturation of the retina. Light-adapted and flicker electroretinograms (ERGs) show responses of the cone system, whereas dark-adapted ERGs primarily reflect the function of the rod system. Cones develop earlier in gestation, but mature more slowly than rods [6]. With sufficiently strong stimulation, ERGs can be recorded in very preterm children with GA < 31 weeks [7–9], although retinal sensitivity is reduced in preterm infants relative to that of full-term infants. Increasing amplitudes of light-adapted and flicker ERGs within the first months of life show the increase in retinal responsivity and reflect rapid physiological maturation of the cone system of the retina during this time [7, 10–12]. In dark-adapted ERGs of preterm infants, retinal sensitivity increased by 1.5 log units and retinal responsivity showed a 4-fold increase between 30 and 40 weeks postmenstrual age (PMA) [7]. Abnormalities of the rod system in infants with ROP have been reported, such as diminished retinal sensitivity in dark-adapted ERGs [9] or prolonged maturation of thresholds in comparison to infants without ROP [13]. In addition, a transient loss of retinal sensitivity between 34 and 36 weeks PMA, associated with the onset and resolution of stage II ROP, was reported [12]. There are indications that rods are more affected by ROP and that deficits in cone function are limited to infants suffering from severe ROP [14]. However, flicker ERG abnormalities have been suggested as a potential early indicator of severe ROP, since two infants who later developed ROP requiring treatment had diminished flicker ERG amplitudes at 36 weeks PMA in comparison to infants without or with mild ROP [9].

As untreated ROP can lead to visual impairment and blindness, infants at risk for ROP require standardised and repeated retinal examinations including dilated fundoscopy at appropriate intervals by experienced paediatric ophthalmologists [15]. Local guidelines recommend screening severely preterm infants with a GA < 30–31 weeks and/or with birth weight <1500 g. In addition to low gestational age and low birth weight, other risk factors for ROP include hypotension, hyper- and hypoxia, suboptimal nutrition, and infections [16–18]. The presence of such risk factors can necessitate screening in more mature (less preterm) and larger children [15, 19, 20]. Advances in perinatal care have led to higher survival rates of very preterm children, and as a result the number of infants at risk for ROP has increased worldwide [21, 22]. However, there is a lack of screening and treatment resources, especially in low- and middle-income countries, that leads to a relatively high incidence of ROP [21, 23, 24].

The International Society for Clinical Electrophysiology of Vision (ISCEV) defines standard protocols and parameters for clinical ERGs in adults and children. Standard full-field ERG recording requires corneal or comparable electrodes, pupil dilation, periods of dark- and light-adaptation, and open-eye stimulation of the retina. Thus, adapted protocols can be useful, particularly in young children [25]. In addition, the premature retina has low responsivity with small amplitude ERGs which may be non-recordable to ISCEV Standard stimuli so special protocols are required [11, 12]. Recording flicker ERGs with a handheld device and skin electrodes is a non-invasive option to assess retinal dysfunction. ERG recordings with the RETeval® device (LKC Technologies Inc., Gaithersburg MD, USA) can be used without mydriasis in those with open eyes and steady fixation to obtain standard ERGs by adjusting stimulus flashes for pupil size in real time [26]. A recent study [27] demonstrated the feasibility of recording light-adapted ERGs (3.0 and flicker stimulus) in infants born prematurely without ROP using the RETeval® device and skin electrodes with dilated pupils and an eyelid speculum [27].

We recently published a feasibility study showing high success rates for recording good quality flicker ERG waveforms in term born infants through closed eyelids using a non-invasive flicker ERG protocol [28]. A series of strong flashes were used to compensate for the undilated pupils and closed eyelids. The aim of the present study was to use the same protocol to assess retinal function in extremely and moderate preterm infants.

## Methods

### Study design

This study was designed as a non-randomised, cross-sectional single-centre study. ERG data were prospectively collected between November 2020 and November 2022 at the University Hospital Zurich, Switzerland. Written informed consent was given by parents/guardians of all participating children prior to the examination. Data collection and evaluation for this study were approved by the local ethics committee of the Canton of Zurich (Business Administration System for Ethics Committees-Number: 2019-01245).

### Subjects

We collected flicker ERG data from two groups. Group A (healthy moderate preterm infants with GA 34 0/7 to 36 6/7 weeks) was tested in the first week of life. Group B (very and extremely preterm infants with GA ≤ 31 weeks) was tested between the 34th and 37th week PMA. Recordings were performed at the maternity ward (Group A), intermediate care unit (Group B), or at outpatient ROP screening appointments. A reference group of 11 healthy young adults was tested using the same protocol with closed eyelids and natural pupils. Infants participating in the study received standard clinical care including ROP screening when indicated. Based on previous data for flicker ERGs [9] to 10 cd.s.m^−2^ flicker stimulus at 29.4 Hz, we expected to detect a 30% difference in the flicker ERG at the fundamental frequency using sample sizes of 39 full-term infants and healthy preterm infants 34 to 37 weeks gestation (power =0.8, alpha= 0.05, beta=0.2).

### Exclusion criteria

All infants with ocular or systemic malformations, known chromosomal aberrations or significant postnatal complications were excluded. Exclusion criteria were pre-established.

### Flicker ERG recording

Each child was tested on one occasion using the portable RETeval® Complete device running contemporary firmware version 2.9.4 to 2.11.3 and disposable skin electrodes (Myo-Trace® type MT-ASF, Hurev Co. Ltd, Wonju-Si, Korea). The stimulus is delivered by a mini-ganzfeld, which controls the luminance during flicker and excludes background light during stimulation. Ambient light did not exceed 10 cd/m^2^ prior to any measurements, the level beyond which the RETeval will not self-calibrate and no measurements are possible. The flicker stimuli were presented through closed eyelids and undilated pupils while babies were sleeping. Before testing, the infants were swaddled in a blanket and held in a comfortable position. To calm restless infants, a sucrose drop (Algopedol 24%) [29] was given before or during the examination [28]. Monocular measurements of the more easily accessible eye, depending on the baby’s position, were made. Before placing the electrodes, the skin was gently cleaned with skin disinfectant. The Myo-Trace® recording electrodes were the only electrodes commercially available that were physically small enough to be used in extremely preterm infants. The active recording electrode was placed under the inferior orbital rim, the reference electrode at the temporal canthus, and the ground electrode on the frontal area.

The custom flicker ERG protocol is described in our earlier work with term-born infants [28]. Flicker stimuli were pulses of <5 ms duration presented at 28.3 Hz with stimulus levels of 3, 6, 12, 30 and 50 cd•s/m^2^. The stimuli were generated by LEDS of three different wavelengths (long: 621 nm, medium: 530 nm, short: 470 nm) balanced to produce white light with CIE co-ordinates of x = 0.33 and y = 0.33. Device-based proprietary software processed the ERG recordings in real-time. The software’s criteria were based on recording a reproducible signal, specifically the variance of the phase of the signal at the stimulus frequency. Artefact rejection was performed online through a process of wavelet de-noising and calculation of the trimmed mean. Time-domain averaged waveforms were reconstructed over a temporal window of 1024 ms from the stimulus frequency and first eight measurements. Recordings stopped when the phase criterion of the ERG were met or when the maximum recording time of 15 s (5 s for the 50 cd•s/m^2^ stimulus) was reached [28]. Two valid measurements were made for each stimulus level.

### Analysis

Flicker ERG data were saved then exported to a .csv file using RETeval® RFF Extractor Software (LKC Technologies Inc.). This file, containing de-noised voltage and timing data, was imported into Excel (Microsoft Corporation, Redwood WA, USA) for further processing. After verifying the measurements for reproducibility by visually comparing the two overlaid waveforms, the two recordings at each stimulus level were averaged for each patient. Measurements were defined as reproducible when both individual waveforms exhibited visually identifiable peaks and troughs with phase coherence between the two waveforms. The waveforms were qualitatively assessed for reproducibility by two authors independently, with any differences in interpretation resolved by discussion. Non-reproducible waveforms were reported as non-recordable. Outcome measures for analysis were implicit time to the first peak (ms) and peak-to-peak amplitude for each stimulus level. Statistical analysis was performed in SPSS®. As the ERG data were not normally distributed (Shapiro Wilk Test, data not shown), we used the non-parametric Friedman Test to compare the ERG data of infants between different stimulus levels. The Mann-Whitney-U Test was performed to compare outcomes of patient groups.

## Results

Eighty infants were included in this study, with 40 infants each in Groups A and B. Median GA at birth was 250 days (35 5/7 weeks) in Group A and 189 days (27 0/7 weeks) in Group B. At the time of testing both groups had comparable median PMA with 253 days (Group A) and 252 days (Group B). Only one tested child had darkly pigmented skin, therefore we were not able to assess differences between skin colours. Patient characteristics are summarised in Table 1.

**Table 1:** Characteristics of moderate (Group A) and very or extremely (Group B) preterm infants.

	Group A (n = 40)	Group B (n = 40)
Sex		
male	19	18
female	21	22
PMA at birth, median in days (IQR)	250 (241-254)	189 (178-202)
PMA at recording, median in days (IQR)	253 (246-258)	252 (245-256)
ROP at the time of testing	0	8
Stage 1, zone II		6
Stage 2, zone II		1
Stage 3, zone II		1
ROP treatment at the time of testing	0^a^	0

*IQR* interquartile range, *PMA* postmenstrual age.

^a^one infant required treatment after ERG testing.

All preterm infants tolerated the measurements well, although some infants became slightly restless when the strongest flicker stimuli were applied. All except three of the infants tested showed reproducible ERGs to at least one of the five stimulus strengths. At 30 cd•s/m^2^ we observed the highest proportion of infants with reproducible waveforms (69/80 infants). There was no difference in ERG waveform reproducibility between groups (χ^2^ = 3.02, DF 4, *p* = 0.55 Table 2). Both groups showed increasing amplitudes with increasing flicker strength (Friedman Test, *p* < 0.001), while peak times did not differ significantly between stimulus levels (Table 2).

**Table 2:** Flicker ERG amplitudes and peak times of 80 preterm infants.

Stimulus level (cd•s/m2)		Group A (GA: 34 0/7 to 36 6/7 weeks)				Group B (GA: ≤ 31 weeks)			
		n ERG* (%)	median	IQR	CQD	n ERG* (%)	median	IQR	CQD
Peak time (ms)	3	16 (40)	44.3	13.3	0.14	26 (65)	44.8	11	0.13
	6	24 (60)	44.0	5.4	0.06	21 (53)	46.6	7.7	0.09
	12	33 (83)	45.6	11	0.13	28 (70)	46.1	4.4	0.05
	30	35 (88)	43.5	6.1	0.07	34 (85)	42.0	8.8	0.11
	50	32 (80)	43.0	3.9	0.05	31 (78)	41.0	13.8	0.18
Amplitude† (µV)	3	16 (40)	0.66	0.48	0.40	26 (65)	0.90	0.88	0.44
	6	24 (60)	0.93	0.58	0.33	21 (53)	1.17	0.82	0.39
	12	33 (83)	1.10	0.91	0.45	28 (70)	1.31	1.29	0.48
	30	35 (88)	1.47	1.44	0.49	34 (85)	1.61	1.31	0.36
	50	32 (80)	1.55	1.63	0.43	31 (78)	3.01	2.33	0.38

^*^Number of infants with reproducible ERG waveforms in group A (*n* = 40) and group B (*n* = 40).

† Peak-to-peak amplitude of the flicker waveform.

*CQD* coefficient of quartile deviation, *GA* gestational age, *IQR* interquartile range.

**Fig. 1:**
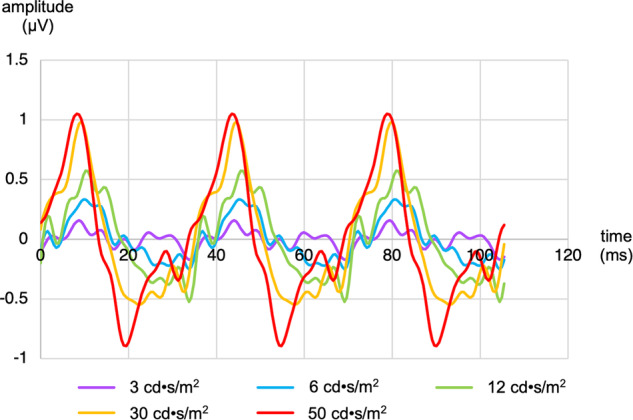
Representative flicker ERG responses of a preterm infant. Five averaged flicker waveforms are shown, corresponding to the five stimulus levels tested.

Figure 1 shows representative waveforms from a preterm infant.

**Fig. 2:**
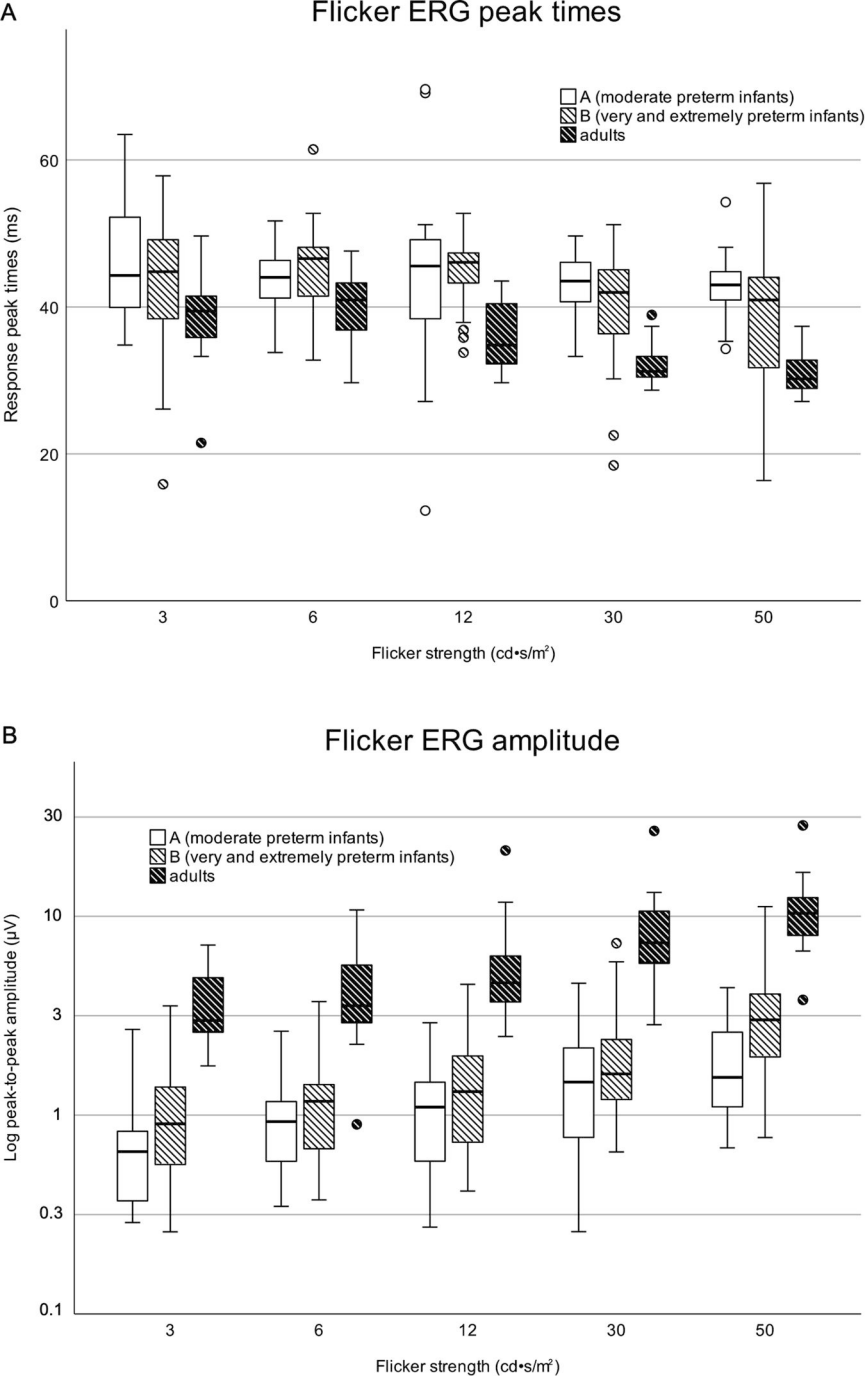
Flicker ERG peak times and amplitudes in infants and healthy adults. Boxplots show flicker ERG implicit times to the first peak (**A**) and flicker ERG amplitudes (**B**) of moderate preterm infants (*n* = 40), very/extremely preterm infants (*n* = 40), and adults with closed eyelids (*n* = 11) for 5 levels of flicker stimuli. Horizontal bars are median values; boxes show interquartile range, capped lines are 95% confidence intervals, dots show statistical outliers.

ERG amplitudes and peak times for each stimulus level of both preterm groups and an adult group, tested with the same ERG protocol through closed eyelids, are plotted in Fig. 2. Group B tended to have higher amplitudes for all stimulus levels than Group A (Table 3), and we confirmed that the effect of Group on the amplitudes was significant (Mean Rank Group A = 122.94, Group B = 158.06; Kruskal-Wallis-H = 13.175; *p* < 0.001). Pairwise comparison of each stimulus level revealed significantly higher amplitudes in Group B only at stimulus level 50 cd•s/m^2^ (Mann-Whitney-U-Test = 198.00, Z = − 4.097, p < 0.001). No inter-group differences were detected in peak times. Adults showed higher amplitudes and shorter peak times than infants for all stimuli. (Table 1, supplementary information.

**Table 3:** Mann–Whitney-U Test.

Stimulus level (cd•s/m2)	3		6		12		30		50	
	peak time	amplitude	peak time	amplitude	peak time	amplitude	peak time	amplitude	peak time	amplitude
Mean rank group A	23.13	16.81	21.13	21.00	31.77	27.33	38.06	31.31	35.88	22.69
Mean rank group B	20.5	24.38	25.14	25.29	30.09	35.32	31.85	38.79	28.00	41.61
Mann-Whitney-U-Test	182	133	207	204	436.5	341	488	198.466	372	198
Z	−0.674	−1.943	−1.025	−1.092	−0.37	−1.75	−1.286	−1.548	−1.707	−4.097
Asymptotic significance (2-tailed)	0.500	0.052	0.305	0.275	0.71	0.08	0.199	0.122	0.088	<0.001*

^*^significant *p* value.

Eight of 40 infants in Group B had ROP when their ERG was recorded, six infants with ROP stage 1, zone II, one infant with ROP stage 2, zone II, and one infant with ROP stage 3, zone II (Table 1). No infant had a ROP requiring treatment nor received any ROP treatment at the time that ERGs were measured. One infant subsequently developed a treatment-requiring ROP Stage 3 zone II with plus disease and received laser coagulation therapy 32 days after ERG testing. Although the number of affected infants was too small to detect and quantify differences associated with ROP, we observed that infants with mild ROP (stage 1) showed a wider range of flicker ERG amplitudes. The two infants with more severe stages of ROP (stage 2 and 3) tended to have higher amplitudes (Fig. 1, supplementary information). One infant with ROP stage 1, zone I, had no reproducible ERG waveforms for any stimulus level. The reason remains unclear to us, and it may be an artefact such as a low signal to noise level, reflect delayed retinal development, or be the result of electrode failure, as the infant’s vision developed well at further clinical check-ups.

## Discussion

In this work, we have demonstrated the feasibility of recording flicker ERGs with skin electrodes in moderately and very or extremely preterm infants without mydriasis through closed eyelids. All infants tolerated the measurements well, in that they did not react in any way that caused alarm to the study team or the parents, and no signs of testing (e.g. skin abrasions) were present afterwards. From previous recordings in term-born infants, we knew that movements of infants during recording led to noisier and less reproducible ERG waveforms [28]. With this in mind, we tried to test the infants in optimal conditions. We recorded ERGs while infants were sleeping, after feeding when possible, and ensured that they were in a comfortable position. However, we had low success rates when trying to test each eye sequentially in term-born infants as exposure to the strongest stimulus and repositioning tended to make infants restless. Reliable testing of both eyes in sequence may require amending the test protocol and omitting the strongest stimulus strength.

Increasing stimulus strength led to higher proportion of ERGs with acceptable waveforms, with the highest proportion for the second strongest stimulus (30 cd•s/m^2^). When the flicker ERG protocol for term-born infants was developed and validated in our previous study, we observed that some infants became restless during the strongest stimulation of 50 cd•s/m^2^ [28]. As a consequence, the maximum recording time at this stimulus level was shortened in the protocol from 15 to five seconds per measurement. It is possible that either the increased likelihood of restlessness of the infants and/or the shorter recording time (with less data for averaging and de-noising) could account for the slightly lower reproducibility at 50 cd•s/m^2^ in comparison to 30 cd•s/m^2^. As the lower stimulus levels led more often to non-reproducible or non-recordable responses, a shortened protocol may potentially offer a valuable measure of retinal function whilst enabling a shorter examination. The results of a recent study, in which the authors also analysed flicker ERGs in preterm infants using the RETeval® device, cannot be directly compared with ours due to use of different ERG protocols and inclusion criteria [27].

We could acquire visibly reproducible ERG waveforms at least in one stimulus level in most preterm infants. However, in three infants no reproducible ERGs were detectable to any stimulus. In one of these infants, we observed intermittently opened eyes and high electrode impedance that likely led to poor recording quality. In the other two infants, no obvious abnormalities during recording were noted. In comparison to the group of term-born infants described previously [28], these preterm infants had a lower rates of reproducible ERGs, although for the optimal flicker stimulus, success rates of 88% and 85% were achieved for the moderate and very/extremely preterm infants, respectively. Due to the physically small size of the premature babies’ faces and the frequent use of nasal feeding tubes or continuous positive airway pressure (CPAP) ventilation, electrode placement was often challenging, even with small skin electrodes. The skin electrodes we used did not always adhere optimally to the skin and sometimes detached during recordings, necessitating reattachment. Another factor is the highly sensitive and fragile nature of the skin in preterm infants, which allowed only gentle wiping with skin disinfectant and precluded the meticulous skin preparation and scrubbing with abrasive paste usually used prior to ERG recording. Special electrodes designed for recording ERG in children and babies were not available at the time of recording; since then a new commercial product has become available (Small size RETeval Sensorstrip®) and new electrode designs with increased flexibility and conductivity show promise [30]. In summary, whilst those non-recordable and non-reproducible flicker ERGs documented here may be partly due to retinal immaturity with low responsiveness to the weaker stimuli, technical reasons likely also contribute. Improvements in skin electrodes may enable more reproducible measurements in the future.

Although both groups had similar PMAs at the time of testing, very and extremely preterm infants tended to have higher amplitudes than moderate preterm infants across the whole stimulus range, with a significant p-value at the strongest stimulus level of 50 cd•s/m^2^. This indicates that extreme prematurity is not associated with reduced retinal responses to flicker stimuli. The higher flicker ERG amplitude in very and extremely preterm infants could suggest a higher retinal responsivity in the observed period between 34-37 weeks PMA, although we cannot rule out interventions such as supplemental oxygen as contributors to the higher amplitudes. An earlier study reported improved sensitivity in dark-adapted ERGs in extremely premature infants compared with term-born infants at when both were tested at term-age. This difference was no longer detectable at 50 weeks PMA [7]. Our results also support these authors’ suggestion that exposure to extrauterine illumination has a triggering effect and therefore accelerates retinal development after preterm birth.

Although our study was not designed and powered to detect significant differences between infants with and without ROP, we observed that the two infants with higher ROP stages had supernormal flicker ERG amplitudes. A pilot study in our centre that tested a small number of infants with ROP using the same flicker ERG protocol showed similar results, with higher amplitudes in infants with treatment-requiring ROP [31]. Supernormal flicker ERGs might be caused by changes in electrical activity of retinal cells due to a hypoxia driven increase of anti-VEGF levels in the retinal tissue [32]. Nevertheless, the findings could be coincidental, particularly as we had a very small number of infants affected by ROP, reflecting the low incidence of ROP at our centre [22]. We encourage further studies assessing larger cohorts of infants with ROP using our protocol. Also, prospective longitudinal data will be required to show to establish the time course for development and to show any differences in flicker ERGs with ROP.

The eyelids of new-born infants transmit up to 38% more light than those of adults, although only at longer wavelengths (due to the red-pass filtering properties of the eyelids) [33]. This difference in transmission is hypothesised to be primarily due to thinner eyelids in neonates [33]. Conversely, retinal sensitivity is relatively low in infants [6]. Our data confirm that reproducible flicker ERGs to the same stimuli can be expected in both preterm infants and adults, albeit with higher amplitudes and shorter peak times in adult ERGs.

In summary, we have shown that non-invasive flicker ERG recordings in preterm infants are feasible, although may be less reliable than in adults and term-born infants. The reasons for the observed lower reproducibility in preterm than in term-born infants are most likely in technical nature. Recent developments in electrode design are promising, and further studies with smaller skin electrodes more suitable for use with pre-term infants should be considered. A limitation of our flicker ERG protocol is that only cone pathway function is assessed, even though rod system function is known to be affected by ROP. We plan to investigate our protocol as an additional tool to assess infants at risk for treatment-requiring ROP. The easy handling of the device and the non-invasive nature of the ERG testing represent clear advantages of this method, and will enable not only ophthalmologists but also trained nurses/technicians to perform the examination. Finally, more infants with ROP need to be tested in further studies to evaluate maturation of the cone system of the retina in preterm infants with and without ROP.

## Summary

### What was known before

- Electroretinography reflects maturation of the premature retina. Infants affected by retinopathy of prematurity have shown abnormalities in electroretinograms (ERGs).
- Feasibility of a novel non-invasive flicker ERG protocol has been demonstrated in a cohort of term-born infants.

### What this study adds

- Feasibility of non-invasive flicker ERG recording with skin electrodes through closed eyelids in preterm infants was shown.
- Extremely premature birth was not associated with diminished or delayed flicker ERGs. Very and extremely preterm infants tended to have higher flicker ERG amplitudes than moderate preterm infants, but with significantly higher amplitudes only at the strongest stimulus level.

## Supplementary information

Figure 1, supplementary information

Table 1, supplementary information

## Data Availability

The datasets generated during and/or analysed during the current study are available from the corresponding author on reasonable request.
