# Supplementary figures and images for: Flicker electroretinogram in preterm infants
Source: Eye (Lond). 2024 May 23;38(14):2768–74. doi: 10.1038/s41433-024-03127-9 (PMC11427446; doi:10.1038/s41433-024-03127-9)

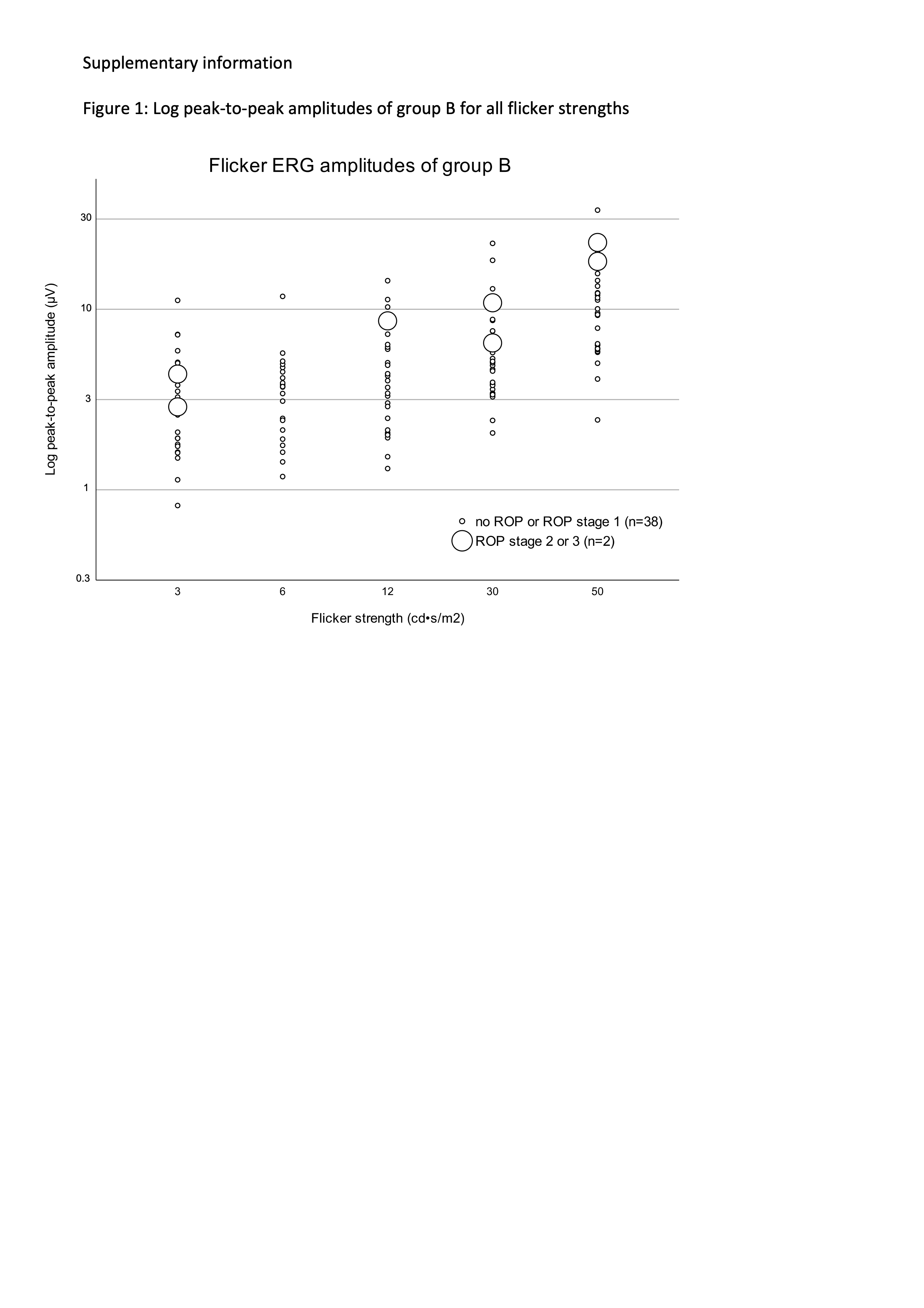

Supplement: Supplementary file 1 — Figure 1, supplementary information [file 41433_2024_3127_MOESM1_ESM.tif]
