# Supplementary material for: Flicker electroretinogram in preterm infants
Source: Eye (Lond). 2024 May 23;38(14):2768–74. doi: 10.1038/s41433-024-03127-9 (PMC11427446; doi:10.1038/s41433-024-03127-9)
Supplement: Supplementary file 2 — Table 1, supplementary information [file 41433_2024_3127_MOESM2_ESM.docx]

**Supplementary information**

**Table 1**: Flicker ERG data collected through closed eyelids of 11 healthy adults

| stimulus  level  (cd•s/m^2^) | | **adults (n=11)** |
| --- | --- | --- |
|  |  | median (IQR) |
| **Peak time (ms)** | 3 | 39.4 (34.3-42.0) |
|  | 6 | 41.0 (36.9-43.5) |
|  | 12 | 34.8 (31.7-41.0) |
|  | 30 | 31.2 (29.7-33.3) |
|  | 50 | 30.2 (28.7-32.8) |
| **Amplitude (µV)** | 3 | 3.0 (1.9-5.1) |
|  | 6 | 3.4 (2.3-5.0) |
|  | 12 | 4.6 (3.6-7.6) |
|  | 30 | 7.4 (5.7-11.1) |
|  | 50 | 10.4 (7.0-13.0) |

IQR, interquartile range
